# Highly Soluble Fluorinated Polyimides Synthesized with Hydrothermal Process towards Sustainable Green Technology

**DOI:** 10.3390/polym13213824

**Published:** 2021-11-05

**Authors:** Juheon Lee, Seungho Baek, Jinsu Kim, Sangrae Lee, Jinyoung Kim, Haksoo Han

**Affiliations:** Department of Chemical and Biomolecular Engineering, Yonsei University, 50 Yonsei-ro, Seodaemun-gu, Seoul 03722, Korea; wngjs92@hanmail.net (J.L.); jelicas@naver.com (S.B.); jinsu.kim@yonsei.ac.kr (J.K.); daniel2000alpha@gmail.com (S.L.)

**Keywords:** fluorinated polyimide, hydrothermal process, green

## Abstract

Polyimides, a widely used engineering plastic, require use of large amounts of toxic and hazardous organic solvents which threaten our daily lives, calling for new and easy synthetic methods for sustainable environmentally friendly development. In this paper, highly soluble fluorinated polyimides based on 4,4′-(hexafluoroisopropylidene) diphthalic anhydride were synthesized via hydrothermal process without using any toxic organic solvents and the advantages of the newly demonstrated synthetic methods are shown by comparative analysis performed with the two conventional synthetic methods using organic solvent: thermal and chemical imidization. Lower temperature is required (~200 °C) compared to thermal imidization and functional groups for high fusibility formed more easily compared to chemical imidization. According to the comparative analysis, hydrothermally synthesized PIs showed excellent solubility and maintained high thermal stability (>500 °C) and glass transition temperature (>300 °C) compared to conventional PI. The hydrothermally synthesized polyimide is much more convenient to store and manage than other form of polyimide which is much more stable when it is exposed to humidity as it is a powder form. The hydrothermal synthetic method is verified to be a “Green” and facile method for sustainable PI synthesis.

## 1. Introduction

Aromatic polyimides (PIs) are the most valuable super engineering polymer with its excellent thermal stability, mechanical properties, insulating ability and chemical resistance. Due to those excellent characteristics, they are widely applied and studied in high-tech industries [1,2,3,4]. The most typical application of PIs is the semiconductor and aerospace field as an insulator, due to their thermal resistance to the extreme condition; they are also utilized as a substrate or as cover window for a device with their flexibility and excellent mechanical strength [5,6,7]. In addition, the structure of PI is usually modified for various target properties by controlling or functionalizing the monomers. Especially, fluorinated PIs have attracted a lot of attention owing to their ability to decrease dielectric constant by reducing the dipolar moment and formation of free volume in the polymer chain and they have many advantages over optical material application due to their colorless form, with the low charge transfer complex (CTC) of PIs controlled [8,9]. The CTC is a phenomenon in which nitrogen atoms act as electron donors and carbonyl groups act as electron acceptors in the imide structure. A strong inter and intramolecular attraction is generated by CTC, the excellent characteristics and brown color of PI are expressed. By controlling CTC, various properties such as transparency and solubility can be obtained [10]. Regardless of those attractive properties of PIs, environmental restrictions and their poor processability hinders their further usage in various industries. The need for high-temperature processes and great amount of hazardous organic solvents usage such as N-methyl-2-pyrrolidone (NMP) and dimethylacetamide (DMAc) in the PIs synthesis process still requires further research to develop more sustainable synthetic routes [11,12]. 

Generally, PI is synthesized by a two-step polycondensation. In an organic solvent, tetracarboxylic dianhydride and diamine react to form a precursor, poly(amic-acid) (PAA) by polyaddition, and then the imide ring is cyclized through imidization process [13,14]. There are two typical types of imidization methods: thermal and chemical imidization. Thermal imidization is the simplest and most widely used method; varnish-type PAA is spin-coated and cured above 350 °C to produce a fully imidized PI thin film [15]. Since the temperature required is too high to be reached, in areas where it is difficult to apply such a high-temperature process, chemical imidization is used to lower the process temperature. Chemical imidization is a reaction that imidizes PAA in liquid phase by adding dehydrating agents and catalysts. Because the imidization reaction proceeds in a liquid phase, fluorinated PI with high solubility is often used and is also called soluble PI [16,17]. Chemically imidized PI is precipitated in a solid state, which is a more convenient form for transporting than the liquid form PAA varnish and with better processability. When preparing a thin film by dissolving solid chemically imidized PIs in a solvent, a process temperature corresponding to the boiling point of the solvent is sufficient as the imidization reaction has already been completed. Although the required temperature for thin film production is much lower than that of thermal imidization, still too much additional chemicals are used during the synthesis process and those additives cannot prevent the environmental impact. 

Recently, as the solutions for environmental impact, green synthesis methods with low process temperature have been studied, such as the hydrothermal process of PIs that use water [18]. The hydrothermal process is a widely used method for synthesizing inorganic substances such as zeolite and metal organic frameworks and recent attempts of application on polymer research are continuously increased [19,20,21]. Therein, PI was the first pure polymer apart from inorganic to be studied using the hydrothermal synthetic method [22]. Hydrothermal process of PI is a green and facile method that requires only monomers and water. The tetracarboxylic dianhydride monomer is hydrolyzed to tetracarboxylic acid and the diamine monomer is added to form aryl ammonium. These tetracarboxylic acid and aryl ammonium are monomer salts that work as intermediates of the hydrothermal process of PI. The aqueous solution with the monomer salts dispersed is put into a pressure vessel and autogenous pressure is applied at a temperature higher than the boiling point of water to proceed the polycondensation of monomer salts to fully imidized PI. In the works of Unterlass et al., the mechanism of hydrothermal process of PI was demonstrated and analyzed focusing on the synthesis conditions and morphology [23,24,25]. However, using the hydrothermal process, PI is synthesized in the powder form, rather than a film, which is an easier form for use in industry. In addition, high solubility of PI is essential for shaping particles into film. Therefore, achieving a highly soluble PI via hydrothermal process is demonstrated in this study. In addition, in order to verify the potential of this green technology, properties of hydrothermally synthesized PIs using various monomers are analyzed compared with those of PIs synthesized by general methods. 

**Figure 1:**
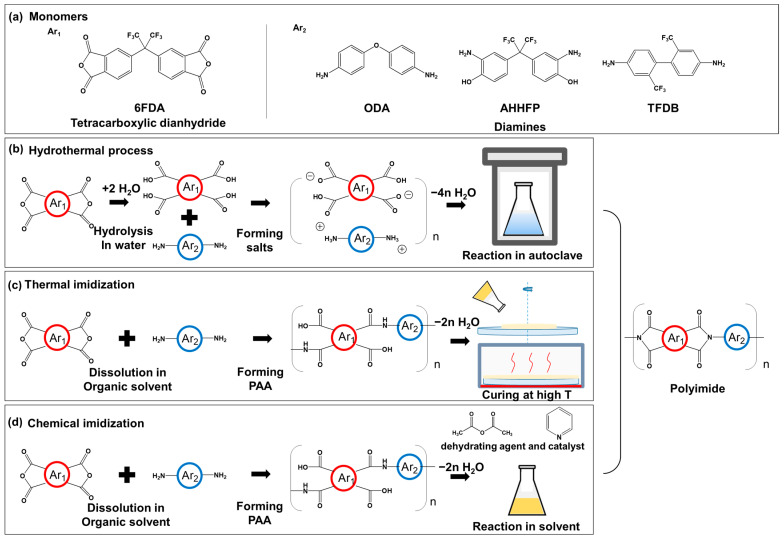
(**a**) monomers for fluorinated polyimides and synthetic pathways of polyimide, (**b**) hydrothermal process, (**c**) thermal imidization, (**d**) chemical imidization.

In this study, highly soluble 4,4′-(hexafluoroisopropylidene) diphthalic anhydride (6FDA)-based fluorinated PIs were synthesized hydrothermally by combining three types of diamine monomers as shown in Figure 1a. 4,4′-Oxydianiline (ODA) is the most common diamine used in Kapton. 2,2-bis(3-amino-4-hydroxyphenyl) hexafluoropropane (AHHFP) has a hydroxyl functional group, so it is often introduced for structural design by grafting PI structure. Bis(trifluoromethyl)benzidine (TFDB) is the best candidate diamine for colorless polyimide due to its symmetry −C(CF_3_)− moiety. To evaluate the industrial applicability of this green synthetic route, it was compared with thermally and chemically imidized PIs. The three kinds of synthetic pathway are described in Figure 1b–d. Thermal imidization is simple but uses toxic organic solvents and requires extremely high temperature condition. Chemical imidization is a process requiring a lower temperature than a thermal one, but more toxic chemicals are involved in the process and they can even induce a side reaction affecting the molecular structures. Combining the benefit of these two methods, the hydrothermal process is expected to bring about a positive effect on the industrial aspect of workability and eco-friendliness.

## 2. Methods

### 2.1. Materials 

4,4′-(hexafluoroisopropylidene) diphthalic anhydride (6FDA, Tokyo Chemical Industry Co., Tokyo, Japan, >98.0%) as a dianhydride and 4,4′-Oxydianiline (ODA, Tokyo Chemical Industry Co., Tokyo, Japan, >98.0%), 2,2-bis(3-amino-4-hydroxyphenyl) hexafluoropropane (AHHFP, Tokyo Chemical Industry Co., Tokyo, Japan, >98.0%), Bis(trifluoromethyl)benzidine (TFDB, Tokyo Chemical Industry Co., Tokyo, Japan, >98.0%) as diamines were used without further purification. For the thermal and chemical imidization, Dimethylacetamide (DMAc, Duksan Chemical Co., >99.0%, Ansan, Korea), acetic anhydride (Duksan Chemical Co., >99.5%, Ansan, Korea) and pyridine (Duksan Chemical Co., >99.5%, Ansan, Korea) were used as purchased. For the hydrothermal process, deionized water (>18 Mohm) was used.

### 2.2. Synthesis of Polyimides via Hydrothermal Process 

For the formation of tetracarboxylic acid, 4 mmol of 6FDA dianhydride monomer was added to 20 mL of deionized water in 30 mL vial and stirred at 80 °C for 2 h. After cooling to room temperature, equimolar diamines according to the molar ratios shown in Table 1 were added in solution. The solution was vigorously stirred overnight and formed white monomer salts. Then, the vial containing the solution was transferred in the bolt-closure-type autoclave of 100 mL in volume. The autoclave was placed in a pre-heated oven at 200 °C for 6 h. After the reaction, the autoclave was slowly cooled to room temperature and opened. The obtained block-like PI powders were filtered and washed with deionized water repeatedly and dried at 80 °C under vacuum for 6 h. Finally, the block-like PI was grinded in a mortar to obtain a yellowish PI powder. The synthetic pathway of hydrothermal process was shown in Figure 1b.

### 2.3. Synthesis of Polyimides via Thermal Imidization

Thermal imidization was conducted in two steps as shown in Figure 1c. First, to synthesize PAA, a precursor of PI, 4 mmol of diamine and 4 mmol of dianhydride were dissolved in DMAc according to the molar ratios shown in Table 1. The solution was stirred at room temperature for 24 h. The concentration of resulting PAA solution was 20 wt%. Then, the solution was spin-coated on a glass substrate for thermal imidization. Thermal imidization was conducted following condition: 100 °C/1 h, 150 °C/0.5 h, 200 °C/0.5 h, 250 °C/0.5 h, 300 °C/0.5 h and 350 °C/0.5 h with heating rate of 2 °C/min. Finally, the thin film PI was obtained.

### 2.4. Synthesis of Polyimides via Chemical Imdization

Chemical imidization was conducted in two steps, as shown in Figure 1d. First, PAA was synthesized in the same way as above. Then, 2 mL each of acetic anhydride and pyridine were added in the precursor solution. The solution was stirred at room temperature for 6 h for chemical imidization. After reaction, the solution was precipitated in deionized water stirred at 300 rpm. The precipitated PI was washed with deionized water and dried at 80 °C for 6 h. Finally, PI fiber was obtained. 

### 2.5. Characterization 

Fourier-transforms infrared (FT-IR) spectra of the polyimides were collected with FT/IR-460 plus (Jasco, Japan) using an attenuated total reflection (ATR) method in the spectral range of 650–4000 cm^−1^. The 400 MHz ^1^H NMR spectra of the hydrothermally synthesized PIs was measured with an AVANCE III HD 400 (Bruker Biospin) spectrometer. The number average molecular weight (Mn), weight average molecular weight (Mw) and polydispersity (PD) were measured using gel permeation chromatography (GPC) Ultimate 3000 (Thermo, Waltham, MA, USA). The thermal degradation temperatures of the monomer salts and PIs were measured by thermogravimetric analyzer (TGA) Q50 (TA Instrument, New Castle, DE, USA) from 35 to 800 °C at a heating rate of 20 °C/min under a nitrogen atmosphere. The glass transition temperatures (T_g_) of the PIs and the heat of reaction of monomer salts were measured using differential scanning calorimetry (DSC) Q10 (TA Instrument, New Castle, DE, USA) from 35 to 350 °C at a heating rate of 10 °C/min under a nitrogen atmosphere.

## 3. Results and Discussion

### 3.1. Polyimide Synthesis

**Figure 2:**
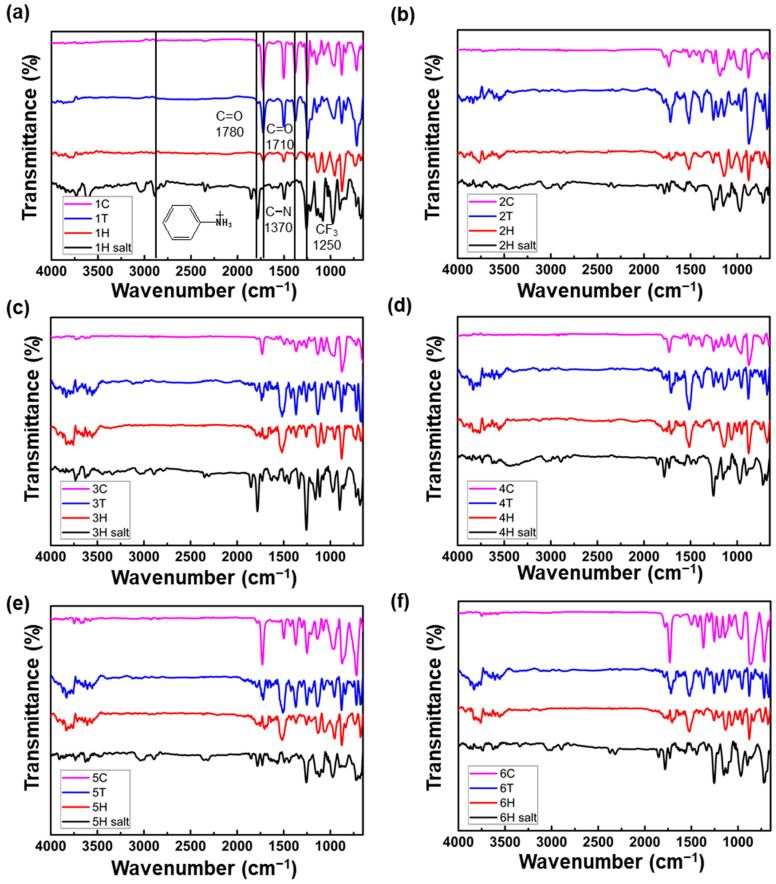
FT-IR spectra of monomer salts and polyimides synthesized with three different synthetic methods, (**a**) polyimide 1, (**b**) polyimide 2, (**c**) polyimide 3, (**d**) polyimide 4, (**e**) polyimide 5, (**f**) polyimide 6.

For structural analysis of hydrothermally synthesized fluorinate PIs, the FT-IR measurement is conducted on the intermediate monomer salts and PIs as shown in Figure 2. The completion of imidization and structural confirmation can be assessed by FT-IR measurement, the representative peaks of imide structure can be observed in following peaks: symmetric C=O stretching 1780 cm^−1^, asymmetric 1720 cm^−1^ and C−N−C stretching 1370 cm^−1^. The peak of trifluoromethyl moiety was also observed at 1250 cm^−1^. As the intermediate of hydrothermal process, the spectra of monomer salts show broad arlyammonium vibration peak at 2830 cm^−1^ and N−H deformation peak at 1473 cm^−1^ [26]. As the tetracarboxylic dianhydride was hydrolyzed to an acid state, it was also shown that the C=O stretching peak was also slightly shifted. According to the Figures S1–S6, showing ^1^H NMR spectra of hydrothermally synthesized PIs, the signals at 7–8.2 ppm indicates aromatic protons of PIs and 10.4 ppm for protons of hydroxyl functional group of AHHFP monomer [27]. 

**Figure 3:**
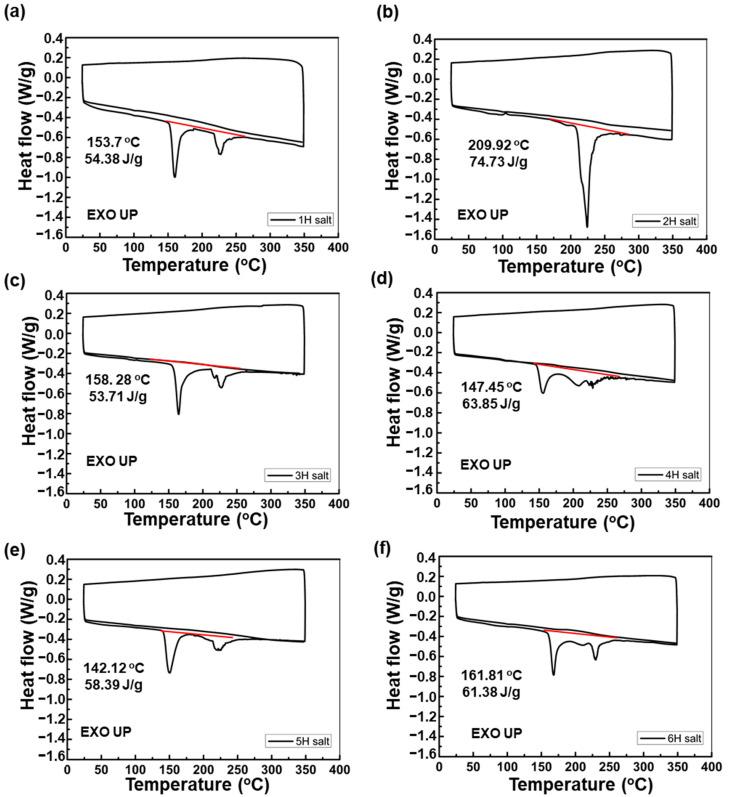
DSC curves of monomer salts which were the intermediate of hydrothermal process, (**a**) monomer salts of 1H, (**b**) monomer salts of 2H, (**c**) monomer salts of 3H, (**d**) monomer salts of 4H, (**e**) monomer salts of 5H, (**f**) monomer salts of 6H.

The DSC measurement of the monomer salts was carried out by three steps: heating, cooling and heating. As shown in Figure 3, all types of PIs showed an endothermic peak occurred only in the first cycle and no specific heat flow such as melting and phase transition was observed in the rest of the cycle. This confirms that only thermally irreversible polymerization occurred. On the other hand, only 2H salt had a single peak and showed the highest heat of reaction of 73.73 J/g and highest polymerization temperature. It is caused by the hydroxyl group in the AHHFP monomer structure indirectly affecting during the imidization process of monomer salts. Basically, imidization of monomer salts was known as a one-step direct reaction, but it has been reported that H_2_O generated during imidization remains in the polymer chain and forms hydrogen bonds with carbonyl moieties, resulting in two-step reaction and two peaks in DSC curves [26,28]. In the case of 2H salt, since hydrogen bonding with H_2_O occurs not only with carbonyl, but also with hydroxyl groups in AHHFP, polymerization needs more energy and occurs at a higher temperature. In the case of 1, 3 and 5H salt without AHHFP monomer, polymerization peak and residual H_2_O removal peak appeared independently, but in 4 and 6H copolyimide salt, the two peaks were connected because the polymerization and H_2_O removal reaction occurred at the same time. In conclusion, 2H salt with a hydroxyl functional group required the most energy and 4, 5 and 6 copolyimides showed approximately the average heat of reaction of PI using each amine alone.

Molecular weight and PDI of the synthesized fluorinated PIs are shown in Table 2 and GPC curves are shown in Figure S9. In all types of PI combinations, the molecular weight of hydrothermally synthesized PI was smaller than that of PI synthesized by the general synthesis methods. However, the PDI was good and the molecular weight was more uniformly distributed. In the case of a general two-step synthesis method, polyaddition takes place in the process of forming PAA by reacting monomers in an organic solvent. Because polymerization occurs in solution, monomers and oligomers move freely and polyaddition occurs irregularly, so the molecular weight is relatively large but lacks uniformity. In the hydrothermal process, the molecular weight is small and shows relatively uniform tendency because solid state polymerization occurs in a closed vessel by temperature and autogenous pressure, which is an external force. 

### 3.2. Thermal Properties 

**Figure 4:**
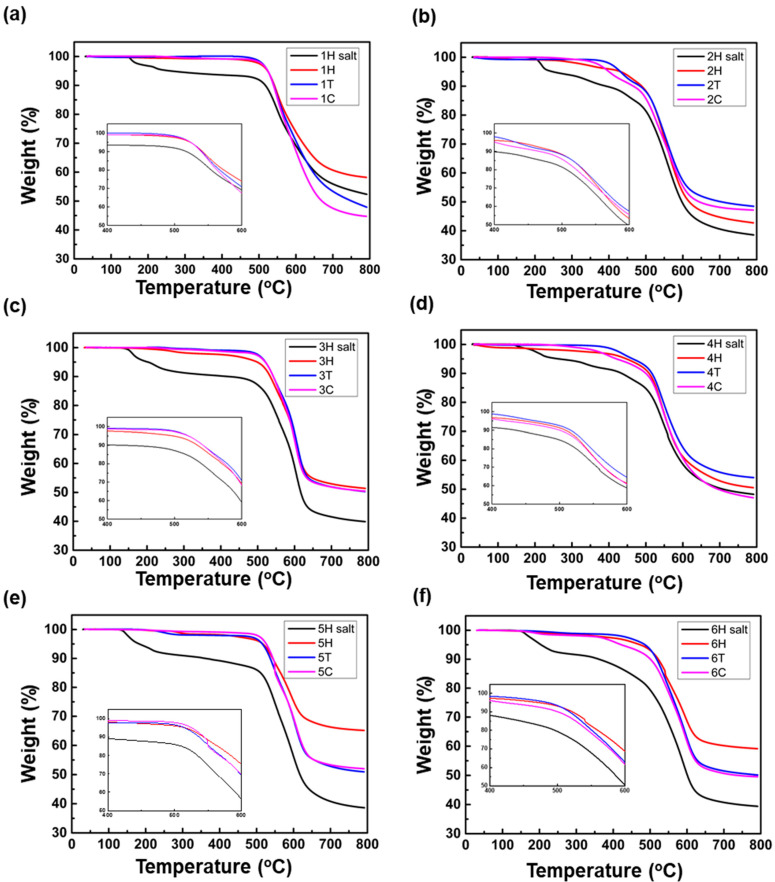
TGA analyses of monomer salts and polyimides synthesized with three different synthetic methods, (**a**) polyimide 1, (**b**) polyimide 2, (**c**) polyimide 3, (**d**) polyimide 4, (**e**) polyimide 5, (**f**) polyimide 6.

The TGA data of monomer salts and PIs are shown in Figure 4. In the TGA graphs of the monomer salts, the weight loss started around 140–200 °C because of the condensed H_2_O molecule during polymerization process. This shows good correspondence with the polymerization temperature of the above DSC data. Thermal degradation temperatures (Td) at 5% and 10% weight loss of PIs are listed in Table 3. The Td of synthesized PI was affected by the structure of diamine and synthesis methods. The Td_5%_ values of the fluorinated PI series show increasing tendency in following order of 2 < 4, 6 < 1, 3, 5. The ODA diamine in PI series No. 1, 4 and 5 formed more stable CTC than AHHFP, TFDB diamine when combined with 6FDA to form PI chains. In general, in the PI structure, a dianhydride containing carbonyl groups acts as an electron acceptor and a diamine with nitrogen atoms act as an electron donor. Since the ether group in the ODA also acted as an electron donor, it was better to form CTCs between the PI chains. So, PI series with ODA had good thermal resistance. AHHFP and TFDB also have CF_3_ moieties with an additional electron donor role, but since the CF_3_ moieties are a bulky structure, they interfered with interchain packing and lower CTC formation [29]. However, since CF_3_ has flame retardant properties, thermal stability was not reduced [30]. AHHFP had the lowest thermal stability because the hydroxyl functional group is relatively weak to heat. 

Meanwhile, in the case of 2, 4, 6C, acetic anhydride added for chemical imidization and some hydroxyl group in AHHFP formed ester groups by acetylation reaction. As ester groups decomposes at 400 °C, lower Td was observed compared to that of PIs synthesized by other synthetic methods. In order to combine with other materials, soluble PI powder is often synthesized using a functionalized monomer such as AHHFP. However, it cannot be synthesized through chemical imidization because functional groups may be damaged. So, PI with specific functional groups that are sensitive to chemical dehydrating agent needs to be synthesized through another synthesis method and a high temperature process in a solution state with additional processes such as reflux system is required [31]. Hydrothermal process has the advantage of being eco-friendly and synthesizing PI easily and with good processability, without affecting functional groups. 

The glass transition temperatures (Tg) of synthesized PIs are listed in Table 3. Tg was obtained from the endothermic inflection point in the DSC curve as shown in Figure S7. In all PI series, thermally imidized PIs showed the highest Tg values. This is because the polymer chain was rearranged as annealing proceeds during the thermal imidization process which leads to the most stable morphology. In the XRD data in Figure S8, it can be confirmed that the synthesized PIs were all amorphous polymer, but relatively sharp peaks appeared in the case of thermal imidization. This indicates that thermally imidized PIs forms a relatively ordered morphology due to the annealing effect and hydrothermally and chemically synthesized PIs with a more amorphous morphologies that are almost like each other. Still, the Tg of hydrothermally synthesized PIs decreased less than 5%. The Tg of chemically imidized PIs also decreased less than 5% for PI series 1, 3 and 5. However, for 2, 4 and 6, side chains were formed in PI structure due to the acetylation reaction as described above, so the Tg was remarkably low. 

Comparing the PI 1–6 series from the aspect of the monomer structure, the linkage structure between the phenyls of the diamine was an important factor the chain mobility [11]. PIs with ODA had a lower Tg owing to the flexible ether linkage of ODA, because the polymer chain moved more easily. The −C(CF_3_)− linkage of AHHFP also facilitated bond rotation and chain movement. TFDB had the most rigid structure because there was no linkage group and had a high Tg value.

### 3.3. Solubility and Processability 

The solubility of PIs was tested with 10 mg of polymer in 1 mL of the solvent. Solvents were selected based on their chemical functional structure: DMAc, NMP as general aprotic solvent, THF as ether, CHCl_3_ as chlorinated solvent, toluene as hydrocarbon, acetone as ketone and ethanol as alcohol. The results of solubility are listed in Table 4. Conventional PIs are known as infusible polymer with poor processability, but synthesized fluorinated PIs had excellent solubility with various organic solvents. Especially, fully fluorinated PIs (2, 3, 6) using AHHFP and TFDB were well dissolved at room temperature in common solvents, such as CHCl_3_ and toluene [32]. In addition, some hydrothermally synthesized PIs were dissolved in ethanol which is known as representative non-solvent of PIs [33]. Since ethanol is widely used non-toxic solvent, it is a favorable candidate for processing the hydrothermally synthesized PIs into film using ethanol as a solvent.

**Figure 5:**
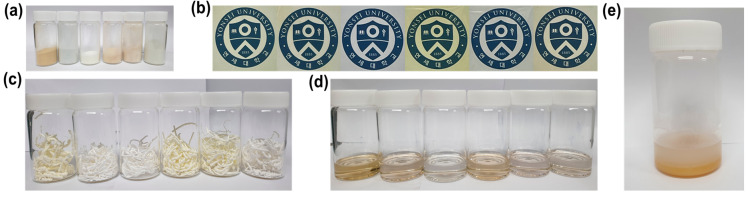
(**a**) polyimide powders in 20 mL vial: resultants of hydrothermal process. (**b**) polyimide films: resultants of thermal imidization. (**c**) polyimide fibers in 70 mL vial: resultants of thermal imidization. (**d**) poly(amic-acid) solution in 70 mL vial. (**e**) poly(amic-acid) damaged by atmospheric moisture in 70 mL vial.

In order to compare the morphology between three different types of PIs, images of synthesized PIs with three different methods are shown in Figure 5a–c, from left to right: PI 1–6. Comparing the colors of synthesized PI, they have similar colors depending on the monomer combination regardless of synthetic method. The PIs containing ODA and AHHFP have a slightly yellowish color and highly colorless polyimide can be synthesized using TFDB with 6FDA. 

Because hydrothermally synthesized PIs have a very fine powder form, their volume is the smallest among the samples, which has great advantages in storage, transport and management. In addition, since PI can be applied in industry by dissolving it in a desired solvent and fabricating a film at low temperature, it has a great potential in industrial application and utilization. Chemically imidized PIs also have a solid fiber form, but with a tough texture, so it is hard to shatter into powder and requires additional process to transform them into a fine powder shape. Thermal imidization is the simplest method to fabricate a PI thin film, but it requires a 350 °C high temperature process and careful management because it should be stored as a PAA precursor solution in liquid form as shown in Figure 5d (from left to right: PI 1–6), which is vulnerable to moisture in the air and chemical structure collapse because of the harsh attack from water molecules transforming into white impurities in Figure 5e. 

## 4. Conclusions

Highly soluble fluorinated PIs were synthesized via hydrothermal process in a much more sustainable way than previous attempts. Instead of using toxic organic solvents, H_2_O was used to synthesize PIs for “Green” synthesis and comparative analysis was performed with conventional synthesis methods. The imidization reaction was observed through structural analysis and proved that fluorinated PIs were synthesized in an environmentally friendly method and without using NMP and DMAc. Creating a water-based soluble PI was first attempted in this study and successfully fabricated, showing a great potential for industrial advantages in storage and handling and making unnecessary the implementation of additional removal facilities for toxic organic solvents. This green synthetic route not only enables sustainable production of PI without using hazardous chemicals, but also greatly reduces the energy required for the process; the temperature needed during the process was reduced to 40% of that of the thermal imidization. Through DSC analysis of intermediate monomer salts, the polymerization of tetracarboxylic acid and aryl ammonium was completed under 200 °C. In addition, the hydrothermal synthesis of PIs has more advantages over chemical imidization, including that it does not require lots of additional chemicals, as in chemical inidization, which causes malfunction of the functional groups in polymer chain with possibility of several unexpected side-reaction. Compared to conventionally synthesized PIs, hydrothermally synthesized PIs showed a lower molecular weight because of solid-state polymerization, but with more uniform molecular weight distribution (PDI < 2) due to the influence of pressure. According to the comparative analysis with conventionally synthesized PIs, the thermal resistance was maintained at over 500 °C and the fusibility was greatly improved in hydrothermally-produced PIs. The PIs with lateral chain functional groups were synthesized without the side reactions that were previously observed with chemical imidization. In conclusion, the hydrothermal process is a sustainable synthetic method with better processability and without adding any additional process or chemicals to achieve maintaining the original thermal and structural properties, which can be the new widely accepted synthetic ways for industries to accomplish sustainable development.

## Figures and Tables

**Table 1:** Molar ratios of fluorinated polyimide and note according to the synthetic methods.

Polyimide	Monomer Molar Ratio				Note ^a^
	6FDA	ODA	AHHFP	TFDB	
1	10	10	-	-	1H, 1T, 1C
2	10	-	10	-	2H, 2T, 2C
3	10	-	-	10	3H, 3T, 3C
4	10	5	5	-	4H, 4T, 4C
5	10	5	-	5	5H, 5T, 5C
6	10	-	5	5	6H, 6T, 6C

^a^ Note hydrothermally synthesized PIs as H, thermally synthesized PIs as T, chemically synthesized PIs as C.

**Table 2:** Molecular weight and polydispersity index of synthesized polyimides.

Polyimide	Synthetic Methods ^a^	Mn ^b^	Mw ^c^	PDI ^d^
1	H	9738	20,781	2.13
	T	9880	47,136	4.77
	C	14,159	41,530	2.93
2	H	6177	12,931	2.09
	T	15,830	130,180	8.22
	C	42,151	99,051	2.35
3	H	7293	10,005	1.37
	T	9381	32,900	3.51
	C	16,442	47,192	2.87
4	H	7397	28,337	3.83
	T	13,953	90,184	6.46
	C	28,339	65,128	2.30
5	H	10,484	21,370	2.04
	T	13,599	48,542	3.57
	C	21,349	62,517	2.93
6	H	7507	14,332	1.91
	T	28,514	78,155	2.74
	C	21,171	71,648	3.38

^a^ Using hydrothermal process note H, thermal imidization T, chemical imidization C. ^b^ Number average molecular weight. ^c^ Weight average molecular weight. ^d^ Polydispersity index (Mw/Mn).

**Table 3:** Thermal properties of synthesized polyimides.

Polyimide	Synthetic Methods ^a^	Td_5%_ ^b^ (°C)	Td_10%_ ^b^ (°C)	Tg ^c^ (°C)
1	H	523.49	541.84	273.63
	T	524.22	540.56	299.98
	C	523.94	539.49	288.21
2	H	432.22	488.97	284.91
	T	431.51	484.58	316.69
	C	400.26	466.32	249.89
3	H	500.22	534.08	322.93
	T	521.25	542.45	324.43
	C	520.49	542.38	312.53
4	H	449.84	508.81	310.93
	T	464.86	516.24	312.82
	C	421.26	500.06	257.17
5	H	517.03	543.74	300.83
	T	515.57	536.94	304.35
	C	526.22	544.41	297.63
6	H	473.07	521.97	301.36
	T	486.07	517.79	317.67
	C	422.13	500.06	256.82

^a^ Using hydrothermal process note H, thermal imidization T, chemical imidization C. ^b^ Degradation temperatures at 5% and 10% weight loss, respectively. ^c^ Reported from second heating thermograms from DSC.

**Table 4:** The qualitative solubility test results of polyimides.

Polyimide	Synthetic Method	Solvent						
		DMAc ^a^	NMP ^b^	THF ^c^	CHCl_3_^d^	Toluene	Acetone	Ethanol
1	H	++	++	++	+	++	++	-
	T	++	++	++	+	+	++	-
	C	++	++	++	+	++	++	-
2	H	++	++	++	++	++	++	+
	T	++	++	++	++	++	++	-
	C	++	++	++	++	++	++	-
3	H	++	++	++	++	++	++	+
	T	++	++	++	++	++	++	-
	C	++	++	++	++	++	++	-
4	H	++	++	++	+	++	++	+
	T	++	++	++	+	++	++	-
	C	++	++	++	+	++	++	-
5	H	++	++	++	+	++	++	-
	T	++	++	++	+	+	++	-
	C	++	++	++	+	++	++	-
6	H	++	++	++	++	++	++	+
	T	++	++	++	++	++	++	-
	C	++	++	++	++	++	++	-

++ soluble at room temperature; + soluble on heating; - insoluble even on heating. ^a^ dimethylacetamide, ^b^ N-methyl-2-pyrrolidone, ^c^ tetrahydrofuran, ^d^ chloroform.

## Data Availability

Not applicable.

## Supplementary Materials

The following are available online at https://www.mdpi.com/article/10.3390/polym13213824/s1, Figure S1: ^1^H NMR of 1H, Figure S2: ^1^H NMR of 2H, Figure S3: ^1^H NMR of 3H, Figure S4: ^1^H NMR of 4H, Figure S5: ^1^H NMR of 5H, Figure S6: ^1^H NMR of 6H, Figure S7: DSC curves of polyimides synthesized with three different methods, Figure S8: XRD patterns of polyimides synthesized with three different methods, Figure S9: GPC curves of polyimides synthesized with three different methods.
