# Supplementary material for: Highly Soluble Fluorinated Polyimides Synthesized with Hydrothermal Process towards Sustainable Green Technology
Source: Polymers (Basel). 2021 Nov 5;13(21):3824. doi: 10.3390/polym13213824 (PMC8587447; doi:10.3390/polym13213824)
Supplement: Supplementary file 1 [file polymers-13-03824-s001.zip › polymers-1427645-supplementary.pdf]

polymers

MDPI

Article

Highly Soluble Fluorinated Polyimides Synthesized with Hydrothermal Process towards Sustainable Green Technology

Juheon Lee, Seungho Baek, Jinsu Kim, Sangrae Lee, Jinyoung Kim *,† and Haksoo Han *,†

Department of Chemical and Biomolecular Engineering, Yonsei University, 50 Yonsei-ro, Seodaemun-gu, Seoul 03722, Korea; wngjs92@hanmail.net (J.L.); jelicas@naver.com (S.B.); jinsu.kim@yonsei.ac.kr (J.K.); daniel2000alpha@gmail.com (S.L.)
* Correspondence: flycorea@naver.com (J.K.); hshanpublication@gmail.com (H.H.)
† These two authors have equally contributed to this study.

Citation: Lee, J.; Baek, S.; Kim, J.; Lee, S.; Kim, J.; Han, H. Highly Soluble Fluorinated Polyimides Synthesized with Hydrothermal Process towards Sustainable Green Technology. *Polymers* **2021**, *13*, 3824. https://doi.org/10.3390/polym13213824

Academic Editor: Andrea Sorrentino

Received: 3 October 2021
Accepted: 3 November 2021
Published: 5 November 2021

Publisher's Note: MDPI stays neutral with regard to jurisdictional claims in published maps and institutional affiliations.

Copyright: © 2021 by the authors. Submitted for possible open access publication under the terms and conditions of the Creative Commons Attribution (CC BY) license (http://creativecommons.org/licenses/by/4.0/).

List of Contents for Supplementary Material:

Figure S1. ^{1}H NMR of 1H.

Figure S2. ^{1}H NMR of 2H.

Figure S3. ^{1}H NMR of 3H.

Figure S4. ^{1}H NMR of 4H.

Figure S5. ^{1}H NMR of 5H.

Figure S6. ^{1}H NMR of 6H.

Figure S7. DSC curves of polyimides synthesized with three different methods.

Figure S8. XRD patterns of polyimides synthesized with three different methods.

Figure S9. GPC curves of polyimides synthesized with three different methods.

SUPPLEMENTARY DATA

^{1}H NMR of hydrothermally synthesized polyimides

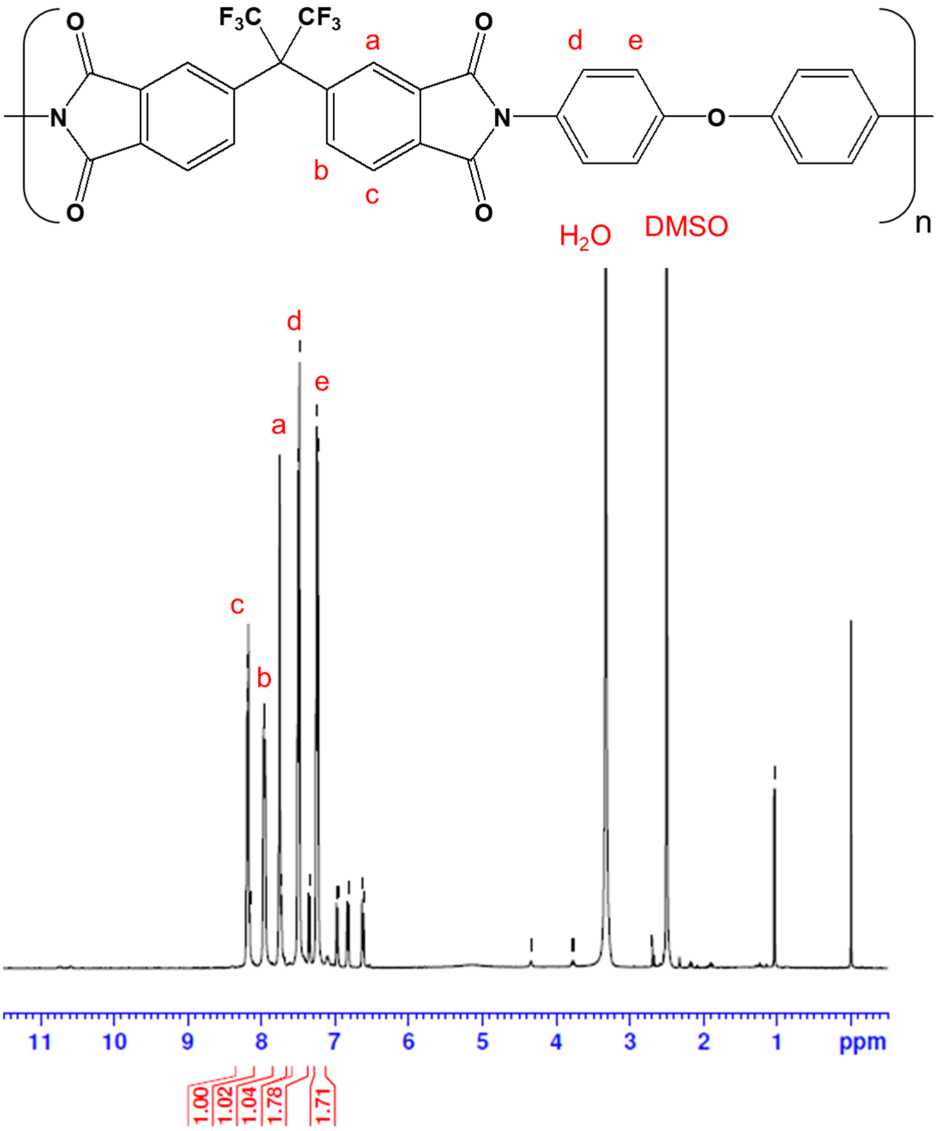

Figure S1. ^{1}H NMR of 1H.

^{1}H NMR (400 MHz, DMSO-d_6): δ=8.17 (2H, Hc), δ=7.96 (2H, Hb), δ=7.49 (2H, Ha), δ=7.35 (4H, Hd), δ=7.24 (4H, He).

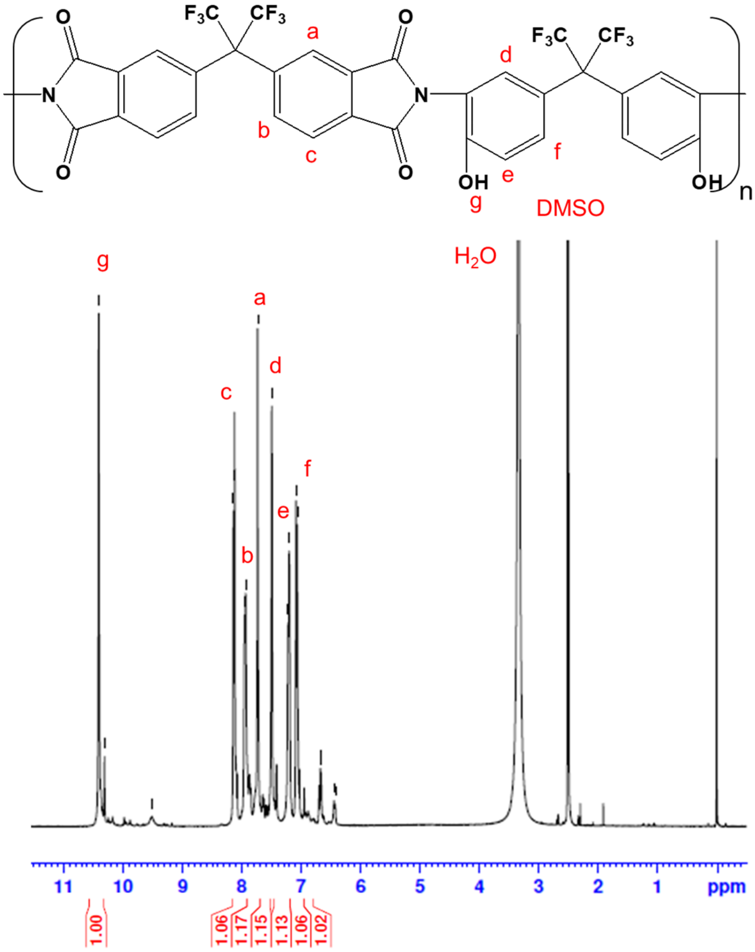

Figure S2. ^{1}H NMR of 2H.

^{1}H NMR (400 MHz, DMSO-d_6): δ=10.41 (2H, Hg), δ=8.13 (2H, Hc), δ=7.94 (2H, Hb), δ=7.73 (2H, Ha), δ=7.49 (2H, Hd), δ=7.21 (2H, He), δ=7.07 (2H, Hf).

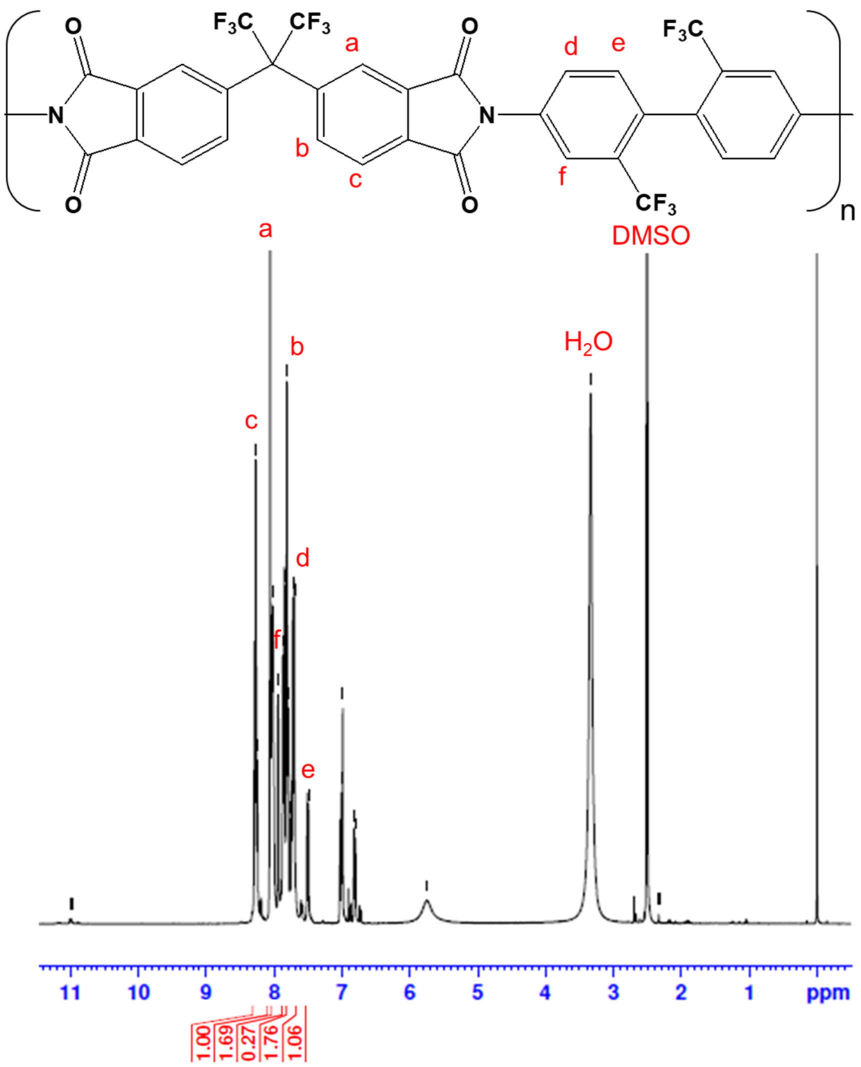

Figure S3. ^{1}H NMR of 3H.

^{1}H NMR (400 MHz, DMSO-d_6): δ=8.25 (2H, Hc), δ=8.03 (2H, Ha), δ=7.94 (2H, Hf), δ=7.76 (2H, Hb), δ=7.72 (2H, Hd), δ=7.51 (2H, He).

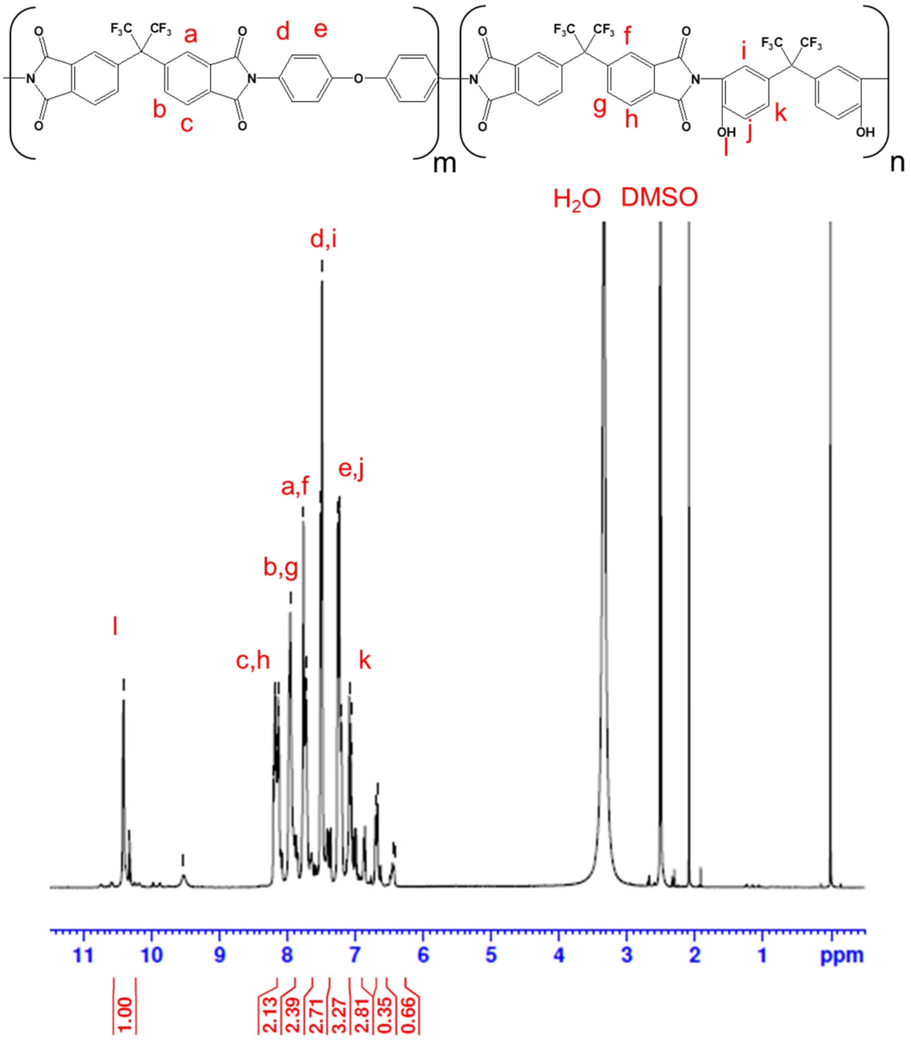

Figure S4. ^{1}H NMR of 4H.

^{1}H NMR (400 MHz, DMSO-d_6): δ=10.41 (2H, Hl), δ=8.18 (2H, Hc), δ=8.15 (2H, Hh), δ=7.95 (2H, Hb), δ=7.91 (2H, Hg), δ=7.76 (2H, Ha), δ=7.73 (2H, Hf), δ=7.51 (4H, Hd), δ=7.49 (2H, Hi), δ=7.25 (4H, He), δ=7.23 (2H, Hj), δ=7.05 (2H, Hk).

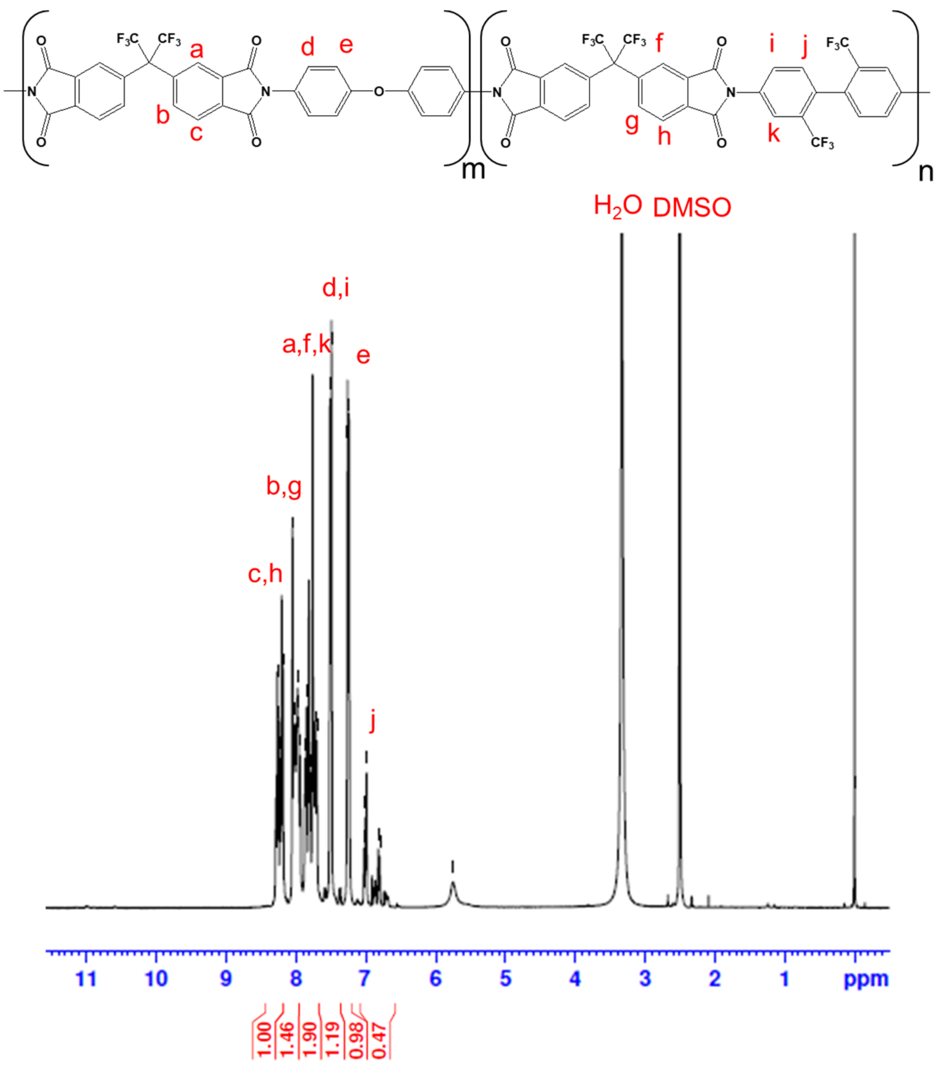

Figure S5. ^{1}H NMR of 5H.

^{1}H NMR (400 MHz, DMSO-d_6): δ=8.26 (2H, Hc), δ=8.22 (2H, Hh), δ=8.05 (2H, Hb), δ=7.95 (2H, Hg), δ=7.85 (2H, Ha), δ=7.81 (2H, Hf), δ=7.79 (2H, Hk), δ=7.51 (4H, Hd), δ=7.49 (2H, Hi), δ=7.23 (4H, He), δ=7.01 (2H, Hj).

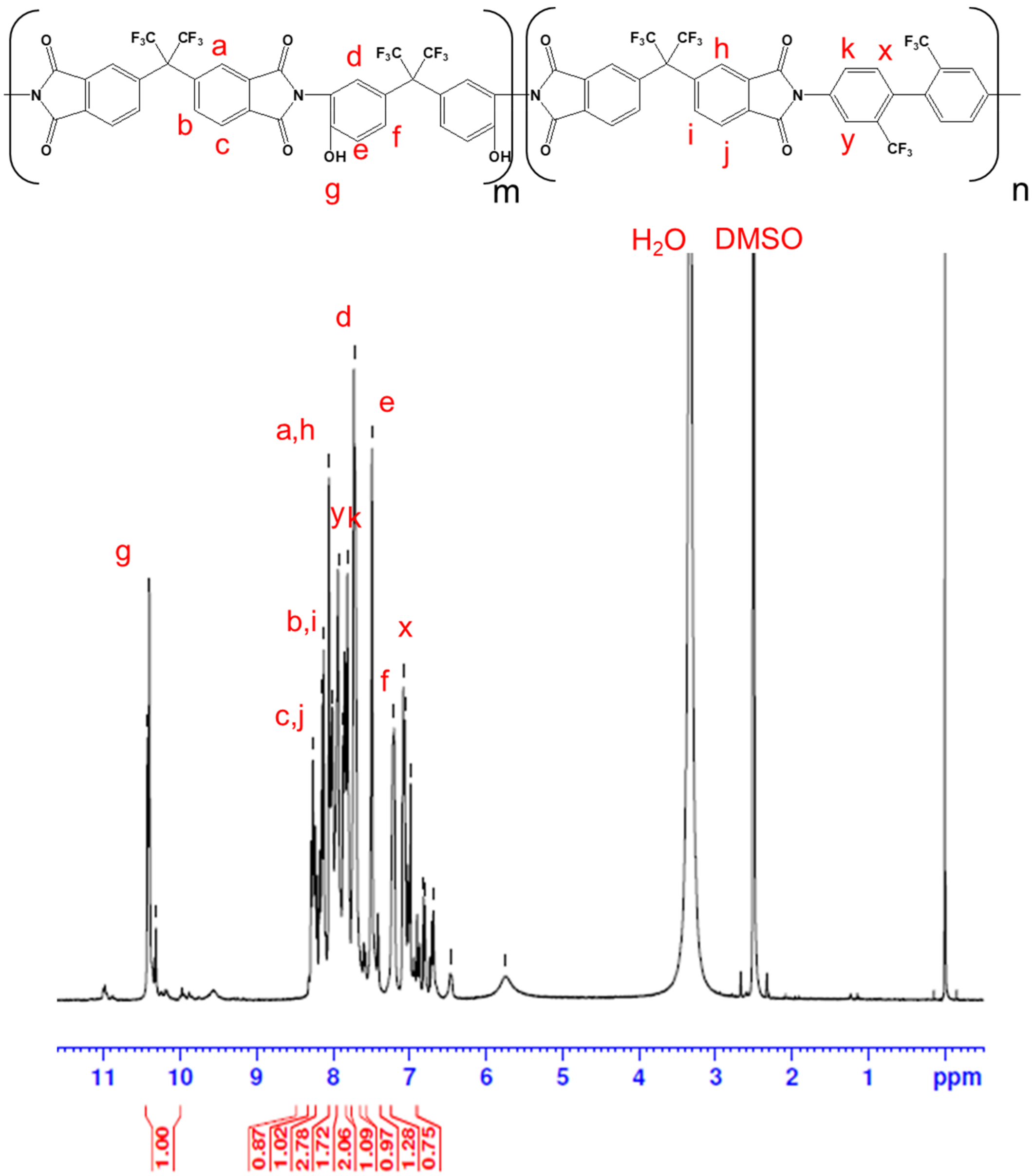

Figure S6. ^1H NMR of 6H.

^1H NMR (400 MHz, DMSO-d_6): δ=10.40 (2H, Hg), δ=8.26 (2H, Hc), δ=8.24 (2H, Hj), δ=8.14 (2H, Hb), δ=8.12 (2H, Hi), δ=8.05 (2H, Ha), δ=7.95 (2H, Hy), δ=7.93 (4H, Hk), δ=7.72 (2H, Hd), δ=7.49 (2H, He), δ=7.21 (2H, Hf), δ=7.08 (2H, Hx).

DSC of synthesized polyimides

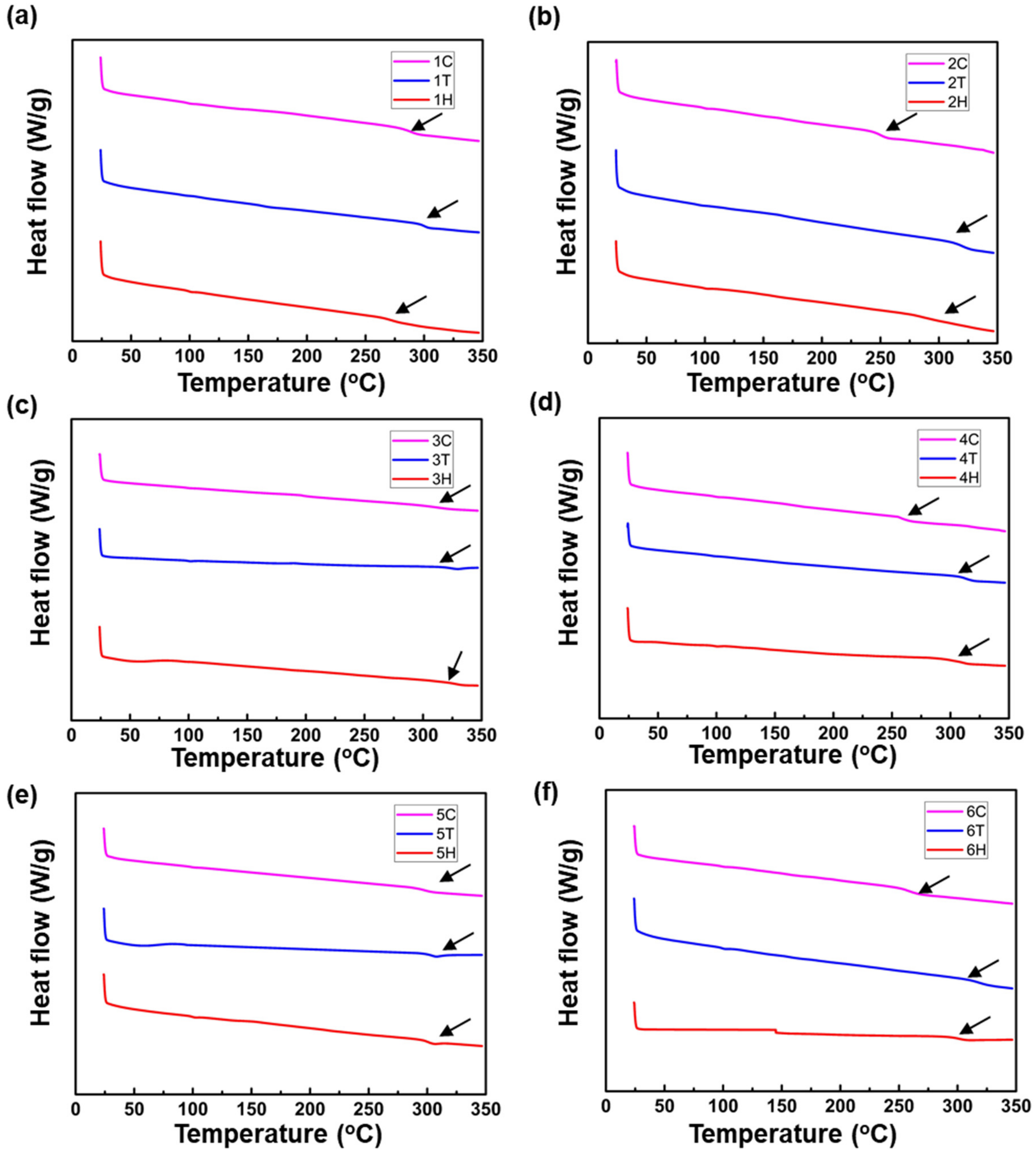

Figure S7. DSC curves of polyimides synthesized with three different methods.

XRD of synthesized polyimides

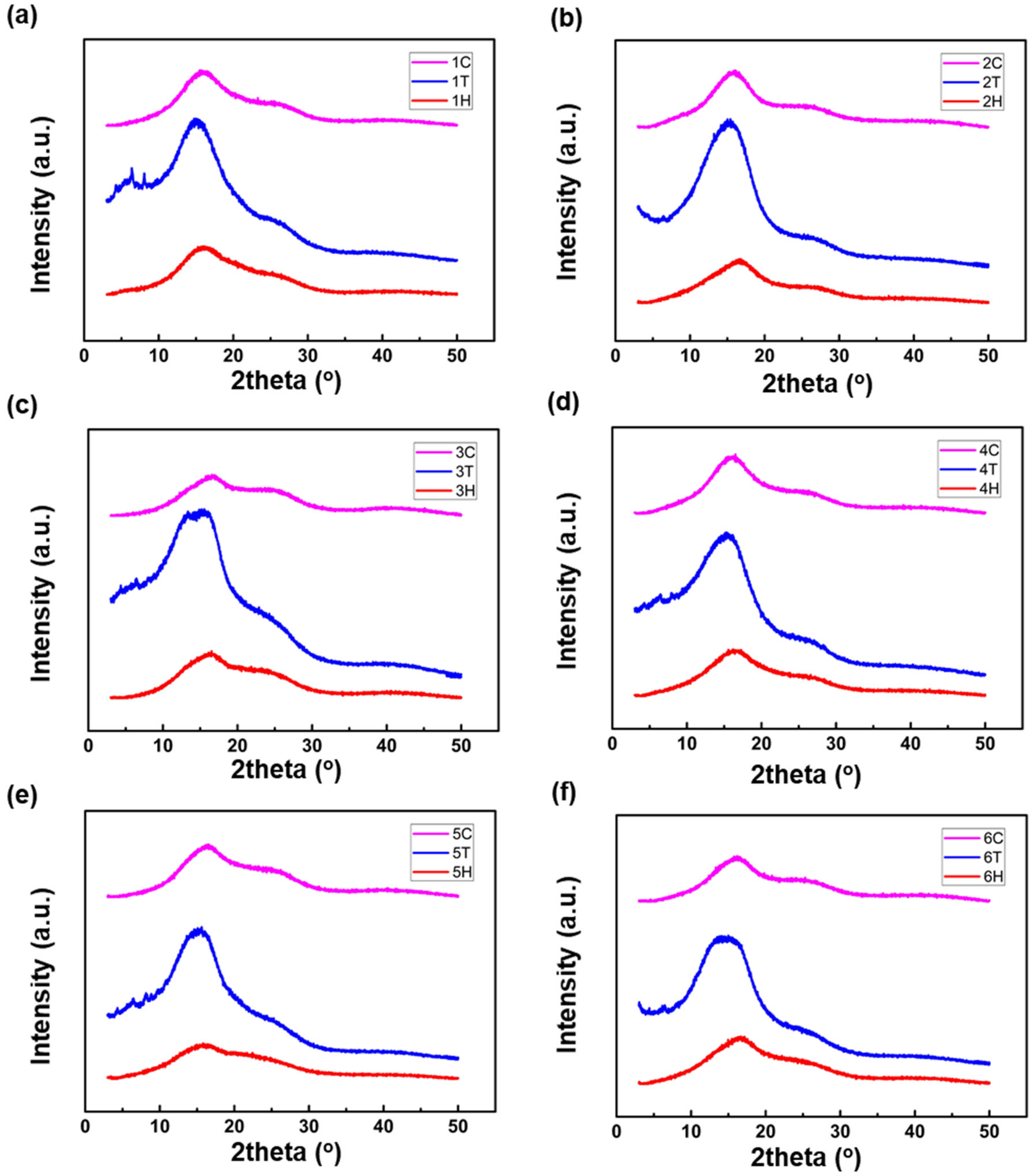

Figure S8. XRD patterns of polyimides synthesized with three different methods.

GPC of synthesized polyimides

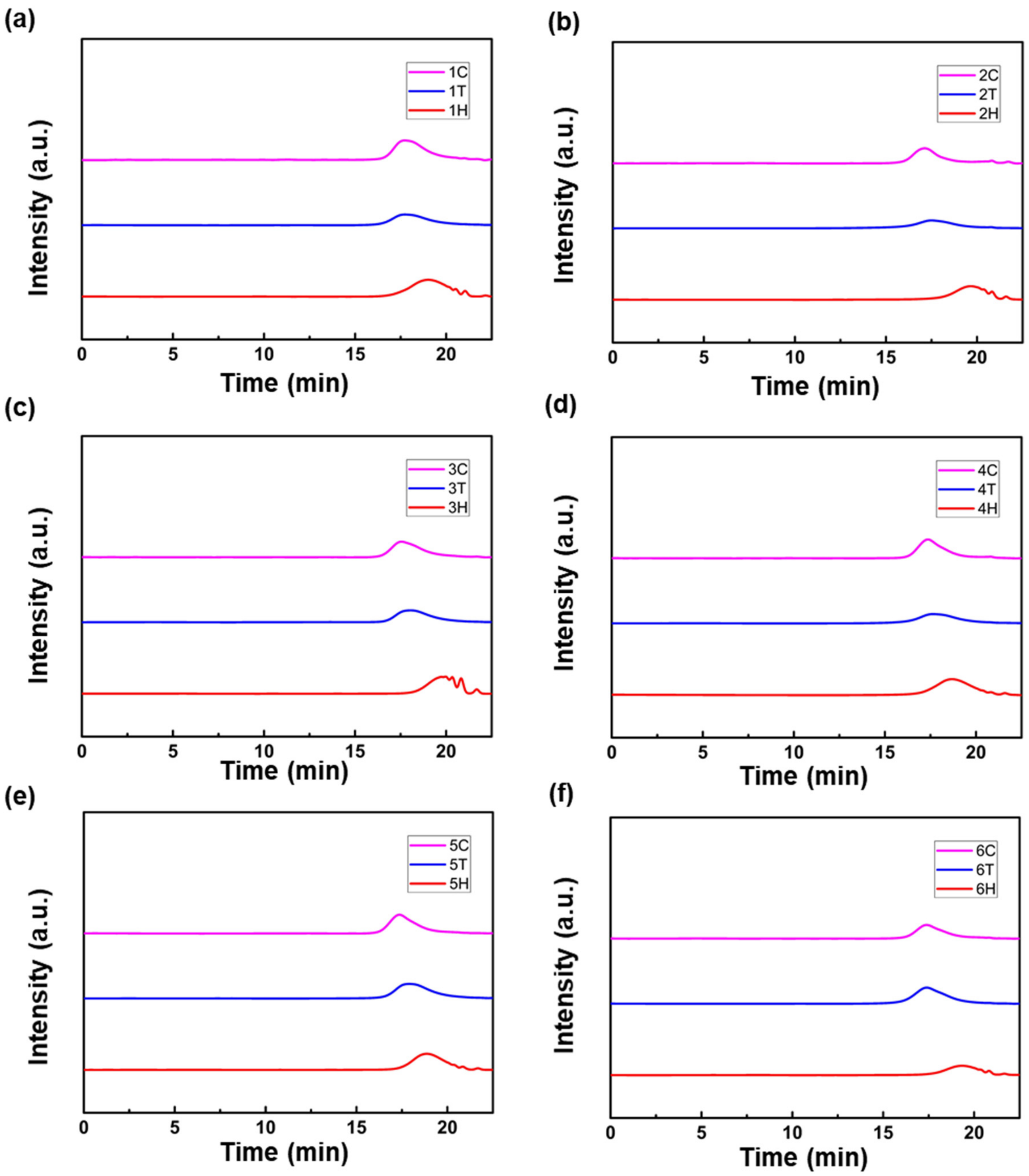

Figure S9. GPC curves of polyimides synthesized with three different methods.
